# Legitimating the Role of a New Profession in a Marketised Healthcare System: The Emergence of Medical Social Work in China

**DOI:** 10.1111/1467-9566.70275

**Published:** 2026-10-21

**Authors:** Haoyang Liu, Paul A. Martin

**Affiliations:** ^1^ School of Sociology Nankai University Tianjin China; ^2^ Guangzhou Digital Technology Group Guangzhou China; ^3^ School of Sociological Studies, Politics and International Relations The University of Sheffield Sheffield UK

## Abstract

This article examines the emergence and legitimation of medical social work (MSW) within China's recently marketised healthcare system, building on longstanding sociological debates about professional formation and authority. Drawing from qualitative interviews with medical social workers, doctors and patients across diverse hospital settings, the study compares two organisational models—internal embedding of MSW within hospitals and external collaboration via government agencies—to analyse strategies for gaining professional legitimacy. Three key strategies are identified: external manifestation through symbolic markers (white coat, blue vest), alignment with political imperatives for mediating doctor–patient disputes and resource mobilisation to support vulnerable patients. Findings consider how these three legitimation strategies are enacted in the two organisational contexts and infrastructural access, while externally affiliated strategies enable flexibility, perceived independence and community linkage. The article argues that MSW legitimacy in China emerges not from seeking exclusive control of specialised knowledge but through dynamic negotiation of identity, symbolic authority and responsiveness to policy priorities, providing an empirical case of post‐professionalism under conditions of market reform. These insights contribute to the sociology of health and illness by highlighting context‐sensitive processes of professional formation in transitional healthcare systems in the Global South.

## 1 Introduction

The ways in which professional groups within healthcare claim and negotiate professional legitimacy have been a longstanding topic of inquiry within sociology (Freidson 1970, 2001; Suchman 1995). Most is known about the profession of medicine and its sub‐disciplines in both marketised (e.g., the United States) and welfare systems (e.g., Europe), mainly in high‐income Global North countries. Here, one of the foundations of professional authority and legitimacy is a client‐centred ideology where doctors are seen to act in the best interests of patients (Niu and Liu 2021). Less is known about the strategies healthcare professions use to claim professional legitimacy in low‐ and middle‐income countries in the Global South or in systems where trust in professions is weak or has broken down as a result of systemic change, such as marketisation.

China provides an excellent site to study the latter as it has undergone a dramatic transition from a planned to a market economy in the 1980s, resulting in a breakdown of doctor–patient relations and growing inequalities in access (Tao et al. 2020; Shi 2022). As the government withdrew from direct service provision, healthcare became profit‐driven, with hospitals raising revenue through increased drug prices and extra services. Since then, the proportion of personal health expenditure paid out‐of‐pocket surged from 20% in the 1980s to approximately 60% by the 2000s, making medical expenses the third‐largest component of residents' personal expenses, after food and housing (National Bureau of Statistics of China 2024). This shift has fuelled public distrust of the medical profession and escalating tension among doctors and patients (Niu and Liu 2021; He and Qian 2016; The Lancet 2020). In response, Chinese health policymakers declared the ‘failures and mistakes’ of the comprehensive market‐driven healthcare reforms, reasserting their steering role through a strategic pivot towards social governance. Within this state‐led framework, medical social work (MSW) has been gradually reinstated in Chinese general hospitals since the 2000s, with the dual mission of mediating doctor–patient disputes, reducing conflict and promoting more equitable access to healthcare services (Liu 2008; Zhao et al. 2018).

Within this broader context, the development of the MSW profession in China can be seen as part of a systemic response to the negative consequences of healthcare marketisation. Initially established to address the growing financial burdens faced by patients, MSW has progressively expanded its role to include the mitigation of healthcare inequalities, particularly through the mobilisation and redistribution of social and material resources for vulnerable groups. Unlike traditional healthcare disciplines rooted firmly in biomedical sciences, MSW operates on a distinct epistemic foundation, grounded in social science, bringing a nonbiomedical lens to healthcare settings and enabling attention to the social determinants and lived experiences of health and illness (Ruth and Marshall 2017). However, as an emerging profession, MSW has confronted an ambiguous professional remit in practice, being perceived as having a less formal status compared with doctors and nurses (Chen and Gong 2019). It has not yet secured public acknowledgement (Y. Zhang et al. 2019). Furthermore, a study by Lei found that doctors frequently treated medical social workers as general administrative staff, reflecting the fragility of their professional legitimacy (Lei et al. 2024).

This study therefore provides an opportunity to understand how the profession seeks to become established in a heavily marketised and rapidly changing healthcare system in a middle‐income country (Meng et al. 2015). To our knowledge, this is the first empirical work to examine the role and function of social work in healthcare teams in the Global South, as well as in the context of healthcare reform. The analysis is enabled by a ‘natural experiment’ given that two distinct models—internal embedding and external collaboration—emerged from the organisation and employment of medical social workers (MSWs). In particular, the paper focuses on the concept of professional legitimacy as a means of better understanding MSWs' work in hospitals, including multidisciplinary case discussion, discharge coordination, safeguarding, psychosocial assessment and patient advocacy that currently constitute the core domains of MSW practice in China (A. Zhang 2019).

This study contributes to the sociology of healthcare professions in three ways. First, by extending existing accounts of the role and formation of MSW (Zheng et al. 2021; Cootes et al. 2022) by providing a detailed qualitative study from contemporary China. Second, it identifies three interrelated sociopolitical strategies which this new professional group has used to claim legitimacy: visibility through external manifestation; alignment with the central state mandates to mediate doctor–patient relationships; and resource mobilisation. Third, it further develops the context‐sensitive approach to understanding professional formation and boundary work and contributes to a more inclusive and reflexive sociology of professions based on incorporating cases from the Global South and healthcare systems in transition. In doing so, the example of Chinese medical social work provides further empirical grounding for the concept of post‐professionalism (Burns 2007), where the legitimacy of an occupational group does not derive from the exclusive control of specialised knowledge, but where professional identity formation is shaped by both market dynamics and political governance.

The first of the following five sections outlines the context of the reemergence of MSW in China and its two development models. The second reviews the literature related to the concept of professional legitimacy in the healthcare field and provides the theoretical background to the study. The third describes the research methods. The fourth presents the empirical findings, respectively, as themes of external professional symbols, fulfilling the central state mandates and resource mobilisation. The final section summarises the findings and discusses their broader implications.

### 1.1 The Development of Medical Social Work in China and the Emergence of Two Employment Models

Medical social work in Western countries emerged in the early 20th century in response to the growing recognition of the ‘social side of illness’, including poverty, housing, overcrowding and immigration (Kreuter et al. 2021). It was subsequently consolidated through professional organisations that established ethical standards and practice guidelines, shaping MSWs' involvement in assessing patients' social and emotional circumstances, facilitating access to resources and promoting holistic, patient‐centred care (BASW 2020).

By contrast, the development of MSW in China, drawing on the values and practices developed in the Western countries, emerged within a distinct sociopolitical and institutional context. Its development took place against the backdrop of market‐oriented healthcare reforms initiated in the 1980s and was explicitly tasked with addressing the growing tensions in doctor–patient relationships. As hospitals became increasingly reliant on self‐generated income, pressures to increase revenue intensified (Yao 2015), and this incentivised doctors, particularly those in public hospitals, to engage in overprescription and the irrational utilisation of services and technologies to ensure hospital viability (Gong et al. 2007; Li and He 2019). At the same time, patient dissatisfaction with healthcare services experienced a marked escalation, concomitant with a dramatic increase in medical disputes. These sometimes became very bitter, involving physical attacks on doctors and even murder, so that 45% of licenced doctors reported experiencing workplace violence in the previous year, with 1% suffering physical‐only attacks and 9% encountering both physical and nonphysical violence (Wu et al. 2021). To tackle this issue, the MSW profession was re‐established in 2006 by the Central Committee and State Council with the aim of improving poor doctor–patient relationships (Liu 2008). Thus, the initial aim for the Chinese MSW profession was to address practical doctor–patient relationship issues. In the process of professional reconstruction, two organisational and employment models have emerged.

#### 1.1.1 The Emergence of Two Employment Models

In 2009, the State Council issued the ‘Opinions on Deepening the Reform of the Medical and Health System’, which explicated the importance of ‘developing MSW, improving the medical dispute resolution mechanism, and enhancing doctor‐patient communication’. To facilitate the recruitment of MSWs and broaden access to MSW services, the General Office of the State Council enacted the Guidelines for Establishing a Modern Hospital Management System. This regulation included ‘having a social work department’ in the hospital annual assessment, encouraging hospital managers to demonstrate compliance with national policy and strengthen dispute mediation and welfare capacities. In many sites, this led to the creation of a model based on ‘internal embedding’ whereby social workers are directly used by hospitals, incorporated into hospital departments and subject to hospital managerial logics (Cheng et al. 2019).

Shortly afterwards, other policies facilitated the emergence of an alternative model based on external collaboration. In some regions where social work agencies were already in operation, the State Council, Ministry of Civil Affairs and other authorities issued the ‘Guiding Opinions on Government Purchase of Services from Social Forces’. This policy enabled local government to procure social work services and provided funding to social work organisations that deploy MSWs to hospitals, without creating new permanent posts within hospital staffing quotas and aligning MSW with existing community‐based social service infrastructures. For instance, in Guangdong province, local government funds MSW services through independent agencies which assign social workers to hospitals. Unlike the internal embedded model, these social workers are used by the government under time‐limited contracts and collaborate with hospitals(Y. Zhang et al. 2019). These represent two principal and practically viable pathways through which local government has sought to operationalise and fulfil central policy objectives in implementation. Since then, the MSW profession has been largely developed around these two distinct models: internal embedding and external collaboration.

Research has explored the roles performed by MSWs, including those of healthcare coordinator, navigator in hospitals and provider of emotional support and patient education (Sun et al. 2023). Some studies have found that MSWs seek interorganisational collaboration, facilitating expertise from diverse sectors to address complex healthcare challenges in a holistic manner (Liu 2008; Sun et al. 2023; Yang et al. 2024). However, most of these publications treat professional standing as an outcome of role performance, without sufficiently interrogating the sociopolitical processes through which professional status is constructed, negotiated and recognised. They also pay little attention to the strategies MSWs in different employment models use to claim professional legitimacy.

Current international social work research indicates social work's foothold in healthcare remains only partially established, owing to insufficient evidence regarding key outcomes and the effectiveness of the profession's knowledge contribution (Matos et al. 2024). An investigation using questionnaires conducted in 1141 hospitals across 26 provinces in China revealed that hospital administrators had limited knowledge of the scope and responsibilities of MSWs, resulting in underutilisation of their expertise and marginalisation within hospitals, with only 21% of them regularly performing their duties (Chen and Gong 2019). Another questionnaire‐based study involving 348 inpatients, randomly selected from eight hospitals providing MSW services, revealed limited awareness of MSWs, with 80% of patients unaware of the profession (Qiu et al. 2016). These findings indicate the low visibility and ambiguous role of MSWs in China's healthcare system. Although such studies offer robust quantitative data, they remain theoretically ungrounded and lack empirically derived insights into the lived experiences of frontline social workers, clinicians and patients they work with.

MSWs in both China and internationally face similar challenges. As Gould (2006) argues, social workers will remain at a distinct disadvantage compared with their healthcare colleagues, and their knowledge risks marginalisation, unless they can clearly articulate their contributions in both integrated care and to public health more broadly. This study empirically examines how MSWs in China are seeking to assert their expertise, redefine their roles and secure systemic recognition. To provide a robust conceptual framework for this analysis, the idea of professional legitimacy will be drawn on and developed.

### 1.2 Professional Legitimacy and Boundary Making

Professional legitimacy is the source of authority for professionals that grants them the right to exercise power in their domain (Abbott 1988). It is the acceptance and trust that professionals and their practices receive from the public, other professions and institutions based on their expertise, qualifications and adherence to ethical standards (Abbott 1988). Suchman (1995, p. 574) defined legitimacy as

a generalized perception or assumption that the actions of an entity are desirable, proper, or appropriate within a socially constructed system of norms, values, beliefs, and definitions.

Professions do not emerge naturally. Classical theories of professional legitimacy emphasise the ways in which professions attain and sustain their privileged status in society through a combination of specialised knowledge, state‐sanctioned jurisdiction, occupational autonomy and a client‐centred ideology (Abbott 1988). In the case of medicine, such knowledge is rooted in biomedical science and clinical expertise, which justifies the profession's exclusive right to diagnose, treat and manage illness. Freidson (1970, 2001) further argued that medicine exemplifies the ‘ideal type’ of a profession because it enjoys state‐sanctioned jurisdiction, which includes regulating its members and defining the standards of its own work, thereby distinguishing it from other occupations. The state, in turn, legitimises this autonomy by granting licensure and regulatory authority to professional bodies. Recent debate highlights the client‐centred ideology that underpins medical professionalism: the idea that professionals act in the best interest of patients, guided by ethical standards rather than market logic or bureaucratic rules (Cao et al. 2023). This ideological commitment to service is seen as a key component of professional legitimacy, reinforcing public trust and justifying the profession's social privileges. Together, these features have traditionally marked healthcare professions, especially in medicine, as legitimate authorities within both the healthcare system and wider society.

Recent theoretical developments have increasingly moved away from viewing professional legitimacy as fixed; instead, legitimacy is now widely understood as a dynamic and context‐sensitive process, shaped by professionals' ability to adapt their roles and identities in response to changing institutional environments (Schot et al. 2020; Tidmarsh 2022a, 2022b). Noordegraaf (2020) argued that contemporary professions increasingly operate under conditions of constrained autonomy, fragmented authority and shifting role expectations. In such settings, legitimacy can no longer be secured by simply asserting exclusive knowledge or claiming jurisdictional control. Instead, professionals must enact new forms of control, demonstrating flexibility, responsiveness and value in context‐specific ways (Timmons et al. 2023). In their institutional analysis of Traditional Chinese Medicine, Tian and Zhang contend that professional legitimacy is shaped by institutional and political factors and clinical efficacy and is acquired through state‐granted and efficacy‐based pathways (2025).

Further debates in contemporary sociology of the professions explore the idea of post‐professionalism (Burns 2007), which Nancarrow and Borthwick (2021) define as ‘the loss of exclusivity over knowledge that is experienced by existing professions’. This is in part a response to rapid technological, organisational and societal change and reflects the growing distribution of knowledge amongst other professional and nonprofessional groups. As a consequence, professions are no longer seen as stable, trait‐based social categories but are an historically contingent and changing set of practices, identities and roles (Burns 2007). Building on Kritzer and developed further by Kaltner et al. (2017), post‐professionalism also entails the formation of new hybrid identities where medicine no longer has total control over health‐related knowledge and practice. In relation to allied health professions, Nancarrow and Borthwick (2021) showed how they take on new tasks and skills that are adopted by other professions to create these novel hybrid identities. Professional legitimacy must instead be actively claimed and negotiated through concrete strategies of boundary work and role performance.

The aforementioned evidence is drawn from studies of shifting roles and jurisdictions between existing healthcare professions, while the case of MSW in China provides an opportunity to study a new healthcare professional group in a rapidly changing marketised healthcare setting in which traditional sources of trust and authority have broken down, leading to a crisis in the doctor–patient relationship.

As outlined earlier, Chinese MSW is currently developing along two trajectories: internal embedding within healthcare organisations responding to market dynamics and external collaboration driven by state initiatives. Initially, the scope and legitimacy of MSWs in China were not explicitly defined by policy, implying that the profession's roles and boundaries were anticipated to evolve organically. This context presents a natural experiment in which the two pathways can be used as contrasting contexts to identify the different strategies pursued by MSWs as they attempt to claim and negotiate professional legitimacy.

In doing this, the paper addresses two main research questions:

1. What strategies have MSWs in China adopted to gain professional legitimacy and how are these enacted in the two employment models?
2. What is the broader implication of this case for understanding the emergence of new professional groups in a marketised welfare system?

## 2 Methods

The interest in this research originated from observing variations in how MSWs were presented in different hospitals; some MSWs wore blue volunteer vests, whereas others were in white uniforms resembling those of doctors. This raised important questions about the role and identity of this newly (re)formed professional group. As a means to investigate, this research used semi‐structured interviews conducted in 2021–2023, obtaining the experiences and perspectives from frontline MSWs, doctors and patients.

This is an exploratory qualitative study based on purposive and snowball sampling. The initial recruitment method involved advertisements on social media. At the same time, three participants were introduced through acquaintances and participated in the interviews. The recruitment process subsequently expanded to include more MSWs, doctors and patients through snowball sampling. Given that the number of MSWs in each hospital is limited, typically two to three individuals, the snowball sampling approach was useful in accessing this limited and dispersed population (Kirchherr and Charles 2018). This also helped to obtain a wide range of perspectives. Overall, a small number of participants were recruited via personal contacts at the start of the study, with most recruited through snowball sampling and the online advert. The study was approved by the University of Sheffield Research Ethics and Integrity (Version 8.3—October 2024). All participants provided informed consent.

There were 55 participants interviewed, coming from 8 regions, covering both economically developed and less‐developed areas in eastern, central and northern China. The sample included 19 public hospitals and 5 private hospitals, reflecting China's healthcare resource distribution in which tertiary public hospitals dominate service provision (Meng et al. 2015). Given the purposive and partly snowball‐based recruitment, the sample is not statistically representative of MSWs, doctors or patients in China. However, the sample covered a wide range of institutional settings, geographical areas and socioeconomic settings. The specific details are presented in Table 1.

**TABLE 1:** Study participants.

Interviewees	Code	Profiles
Medical social workers (*n* = 29)	MSW *n* (1–29)	Medical social workers who had at least two years of clinical‐facing duties or had handled five or more cases in the past year involving communication support, discharge planning, referral, palliative care or dispute mediation.
Doctors (*n* = 13)	D *n* (1–13)	Certificated doctors who have experience working with MSWs.
Patients (*n* = 13)	P *n* (1–13)	Individuals who had multiple healthcare encounters or long‐term chronic disease management experience and had interacted with MSWs.

*Note* *:* Internal MSWs (*n* = 12); external MSWs (*n* = 17).

The MSWs who participated in the study possessed varied levels of experience, ranging from a minimum of two years' experience to over a decade. Four interviewees were among the pioneers of MSW in China, possessing firsthand knowledge and insights into the evolution and development of the MSW profession. To gain further insights and perceptions of MSWs, doctors and patients were purposively selected on the basis that they had experience of engagement with MSWs, which ensured that their views and assessments would provide an authentic representation of the interactions and impact of MSW's services.

Before the interviews, potential participants received a consent form and an information sheet explaining the study's aims and nature. The interviews commenced once the consent form was completed. The semi‐structured interviews were conducted in Mandarin through a hybrid approach that encompassed both online and face‐to‐face methods for the participants' convenience. Although the interviews were open‐ended, the core questions remained. Medical social workers were asked about their professional roles in the hospital, everyday responsibilities and perceived advantages and disadvantages of intervening in doctor–patient relationships. Doctors and patients were invited to describe how they first became aware of MSWs, their experiences of working with or receiving support from MSWs and their perceptions of MSWs' position and authority within the hospital. Interviews lasted between 40 minutes and 2 hours. All sessions were audio‐recorded, with supplementary field notes taken where appropriate. Verbatim transcripts were produced by the first author, usually shortly after each interview, to support early reflection on emerging themes. Sampling continued until thematic saturation was reached, defined as the point at which no new codes or themes were identified in two consecutive interviews (Braun and Clarke 2021). A thematic analysis approach was adopted. This involved a modified ‘constant comparison’ method, where themes emerging from interview data were systematically compared and analysed (Boyatzis 1998). To avoid potential ambiguity or misinterpretation, the original Mandarin used by participants was preserved during the coding process. To ensure reliability and integrity, the researcher continually updated and translated representative content into English and then discussed and reviewed it with the supervisory team. Furthermore, extracts from the participants' responses were directly quoted to maintain the authenticity of the data.

The study initially focused on understanding the role of MSW in doctor–patient relationships. It was hypothesised that MSWs would be perceived as useful by all participants because of their welfare orientation and the support of national policies. However, further research revealed that different employment models produced varying advantages and challenges for MSWs. The resulting framework emphasised three key elements: symbolic power, political backing and control over social resources. This study acknowledges its limitations regarding the representativeness of the participants in reflecting the views of all staff and patients. Rather, it provides a detailed description of the strategies used and a conceptual framework for future, larger‐scale research.

## 3 Findings: Claiming Professional Legitimacy

### 3.1 External Professional Symbols

Medical social workers in the internally embedded and external collaboration models could be identified through their external symbolic presentation. Reported in the interviews, social workers engaged in internally embedded positions in the hospitals always wore white uniforms similar to doctors, namely, the white coat. Conversely, social workers who are engaged through external collaboration within hospitals typically wear a ‘blue vest’. Generally, the choice was determined by hospital regulations; only a few social workers had the option to choose for themselves. It's important to note that the term ‘blue vest’ used in this context is generic; in practice, the colour of the vests worn by MSWs varies among hospitals and may be blue, green, red or other colours, depending on the hospital's requirements. The key point is that the internally embedded model is governed and funded by the hospital itself, while the external collaboration model is contracted and funded by the government. This simplification illustrates the general characteristics of the two models. In some cases, there was overlap or a combination of elements from both models in practice. In the hospital setting, the white coat symbolises medical authority, aligning with patients' cultural respect for healthcare professionals in China (Yao 2015). MSWs, by putting on the same white coat, appear to leverage this symbol to elevate their own professional standing. Those who did so found it easier to build rapport with patients, as the white coat helped bridge initial unfamiliarity and foster smoother communication by associating them with trusted medical staff. The following extract describes how an MSW built a relationship with patients during ward inspections and how the white coat contributed to this.

We do check in the patients as soon as they are inpatients. What I’m wearing is the white coat. For patients, they are unable to distinguish between doctors and medical social workers when we are in the same wearing, they will feel, this guy is from the hospital, and I can believe. (MSW1)

When MSWs in white coats inquired about a patient's condition, the patient and their family showed great willingness in sharing information and an active pursuit of assistance from the MSWs. The white coat represented institutional recognition and acknowledged that these MSWs were part of the hospital. From the patient's perspective, those MSWs could be perceived as representative of the care extended by the hospital or medical professionals and were thus worthy of trust. As a patient said,

I found him in a white coat, I thought, he may be a doctor or someone who works in the hospital, I should be able to trust. (P6)

The internal embedding model allowed MSWs to better focus on their work. However, within the large organisational structures of hospitals, their numbers were very limited compared with the scale of operations involving hundreds of staff and thousands of patients. According to the MSW participants, the smallest departments had only one worker, most had two or three and the largest had a team of four. Such small teams made it difficult to fully carry out their responsibilities. As one MSW noted, they often faced the situation where:

The number in the social work team is too small, too inconspicuous, when we wear white coat, doctors and patients will directly ignore our existence (MSW9).

Dressed in white coats, MSWs were easily mistaken for doctors, which further contributed to them being overshadowed.

Sometimes, my patients called me, ‘doctor’, I will explain I’m not a doctor, I’m social worker. The patients express surprise, ‘I see you many times with doctors’ (MSW4)

In contrast, MSWs who wore ‘blue vests’ faced challenges early on when working in hospitals. Their different attire from the white coats of medical professionals often drew suspicion from patients, as the blue vest does not convey the same authoritative symbol. As a result, patients did not always see them as official hospital staff who could assist with treatment. In fact, two patient participants even mistook the blue‐vest workers for volunteers, assuming that:

I supposed he is a volunteer, there are always volunteers in hospital. (P3) I know he is definitely not a doctor. What I need is a doctor. If he is not a doctor, I think his role in this hospital may not be able to help me. (P5)

In some instances, MSWs even triggered wariness and suspicion among patients and their families. The following extract shows a situation where an MSW in a blue vest noticed a patient's family member crying in the hallway; he went to inquire if he could provide any help. However, this individual exhibited avoidance behaviour and demonstrated a clear reluctance to engage in dialogue or disclose any information to the MSW.

From the patient’s side, it is difficult for him to know what I’m doing if he did not know what medical social work is. It is easy to have some misunderstandings, that is, patients might doubt who I am. That will create difficulties when building the relations. (MSW3)

Further interviews revealed that, although the blue vest initially posed challenges, its distinctiveness helped make the MSW profession more recognisable. Some MSWs wore the blue vest over their white coats, which at first made it easier for others to identify them. As their roles developed, however, they came to see the blue vest as a better expression of their professional identity. Through ongoing efforts, the blue vest has become a symbol of their profession and helped foster public recognition of MSWs.

In fact, I’ve gone through many changes in my career. I wore a white coat at the beginning, but it felt strange because I was not a doctor but dressed like a doctor. It didn’t feel right to me, nor to the doctors, and it confused patients even more. Then I started wearing a vest over the white coat with ‘Medical Social Worker’ written on it. As our department became more stable and recognized in the hospital, we developed our own logo and kept the vest but no longer wore it over the white coat. Looking back, it seems very funny, but through this process, we gradually built our own professional identity. (MSW18)

The distinction between the white coat and the blue vest clearly reflects divergent symbolic strategies for professional positioning among MSWs operating under different institutional arrangements. Rather than mere division of labour, this visual divergence manifests the tension between seeking ‘borrowed legitimacy’ through clinical alignment and asserting an ‘autonomous identity’ through professional differentiation. This difference also brings broader implications. MSWs wearing white coats may find it easier to carry out their work at the start of interventions; the acceptance they receive often stems from the symbolic authority of the white coat, not from the professional legitimacy of MSWs themselves. In contrast, the challenges faced by MSWs in blue vests reflect a broader issue that the identity of MSWs has not been adequately accepted.

### 3.2 Fulfilling the Central State Mandates

The escalation in doctor–patient disputes since the 2000s, due to healthcare reform failures, has promoted the need for third‐party mediation. MSWs were originally introduced to fulfil this role, particularly in conflict prevention. This preventative approach is crucial in establishing the professional legitimacy of MSWs within healthcare settings. As one doctor noted, he first gained a deeper understanding of MSWs when he realised that they could mediate potential disputes; the act of ‘passing’ a difficult case to a social worker signifies a pragmatic acceptance of the profession, where the MSW's mandate is to resolve ‘difficult’ social‐psychological issues that threaten the smooth operation of the clinical environment.

I’m mainly a treatment provider. Besides treatment, I think emotional support is also important, it helps patients recover and prevents medical disputes. But we don’t have the time or energy for that. I first learnt about social workers when a difficult case came up, and since I couldn’t handle it myself, I passed it to our social worker. (D8)

In practice, MSWs focus on patients' lives and emotional experiences alongside their illness. When accompanying the doctor on their rounds, although doctors focused on patients' physical and medical condition, MSWs offer a broader and more accessible account of the patient's situation and function as communication mediators who help patients make sense of complex medical information.

To prevent potential medical disputes, we will go to hospital wards with doctors, record the patient’s condition based on our observations…We will explain their condition, why they are here, the different phases of treatment they may receive and when they could be discharged. We provide a comprehensive overview, which may differ from the doctors' treatment‐focused explanations. Our explanations are closer to daily life, creating positive feelings and impressions, let them feel comfort and care. (MSW1)

As an internal social worker, they hold the view that their work represented the hospital's dedication to delivering superior service and positioned MSWs as representative of the hospital's commitment to preventing conflict.

If patients feel they are overlooked by the hospital, we reassure them of our concern. We emphasise that our services are designed by the hospital to demonstrate our hope for their well‐being and to convey that we genuinely care about them. (MSW1)

Moreover, as hospital employees, internal MSWs cultivate informal relationships with physicians that enable them to identify and address emotional distress arising from medical disputes. They identify doctors' frustrations and provide emotional support, preventing them from developing negative feelings towards patients. Their integration within the hospital enhances their capacity to mediate conflicts and promote organisational stability. A doctor described his experience.

I was really close to getting angry. I’d been trying hard to keep calm. My social work colleague, she knew that I am not happy and talked with me. It works. Being understood was so good, especially in a situation you were in a conflict. (D1)

However, some cases suggested that patients question whether internal MSWs can genuinely represent their interests, as they perceive them as hospital employees. This, in turn, leads to doubts about the internal MSWs' ability to mediate disputes.

I might be conservative about some of them, because I think they are employees of the hospital. They work for the hospital…to what extent can they stand by me? I think, right? (P5)

In contrast, external social workers find it easier to gain patients' trust. As the participant mentioned, MSWs in blue vests are seen as ‘not part of the hospital’. Patients may perceive doctors as having institutional interests, whereas external social workers are seen as more neutral and therefore more trustworthy. This perceived independence facilitates communication and enables MSWs to access patients' concerns more effectively.

…patients sometimes suspect doctors of prescribing costly drugs or using expensive treatment plans. In the ward, they talk to each other and realise they’re being treated for the same illness, some stay only a week and go home, while others stay two weeks and undergo two surgeries. In such cases, we have an advantage. When patients see us in blue vests, they think we’re not part of the hospital, which gives a good impression and makes them more willing to talk to us, a good sign that we have a chance to help resolve the issue. (MSW 28)

Another external social worker noted that they also needed to ‘weigh the pros and cons’ (MSW26), balancing the needs of patients with the interests of the hospital. Completely disregarding the hospital's interests could risk isolation within the hospital. She expressed her concerns as follows:

I can’t always go against the hospital’s requirements; otherwise, they may think I’m not supportive and won’t need me in the future. If I offend the hospital or my doctor colleagues too much, I will be isolated in the future. (MSW26)

A hybrid model has provided a solution to the challenges posed by the two distinct employment models. In an interview with a social worker collaborating with external partners, she explained that their team included MSWs in both internal and external cooperation models. As a result, they have developed a tacit understanding when resolving medical disputes: external social workers focus on comforting patients, whereas internal social workers engage with doctors to understand their perspectives.

We cooperate with the social workers in the hospital. We have a tacit understanding. Also, when there are disputes or complaints, we go to comfort the patients, and my colleagues go to the doctors. (MSW25)

MSWs in both models contribute to meeting the central state mandates by mediating doctor–patient conflicts. However, internally embedded MSWs may face doubts about their neutrality because of their institutional affiliation. This concern is alleviated in the external collaboration model, although external MSWs must avoid appearing overly adversarial towards hospitals and doctors, as this risks marginalisation and undermines their effectiveness.

### 3.3 Resource Mobilisation

Resource mobilisation often involves securing funding and identifying alternative treatment opportunities, thereby enabling individuals who are in challenging situations to access necessary medical care. In a nation marked by vast socioeconomic disparities (Meng et al. 2015), resource mobilisation emerges as a critical function to enhance equitable care access and patient outcomes. The participants reflected on MSWs' professional role in accessing resources and the different strengths of the two models.

Interviews with patients revealed that MSWs were familiar with the hospital's funding opportunities and the complex medical reimbursement procedures, which may be challenging for patients. MSWs have been instrumental in assisting patients to access these funds and resolve financial issues, thereby ensuring that patients can continue their treatment. As one patient said:

A social worker helped me apply for funding…. This significantly reduced my expenses, and I felt a great relief from the financial pressure. At that time, I had almost run out of money for following treatment (P13).

One of the key strengths of MSWs who work internally within hospitals is their deep understanding of the hospital's policies and operational procedures, which position them to act as effective intermediaries between patients and the healthcare system, facilitating access to resources. A social worker described her help in supporting reimbursement:

We’re familiar with the hospital’s reimbursement process. Since regulations differ across hospitals, if a patient asks, we explain the reimbursement details based on our hospital’s policies, including any available funds they might not know about. We will also inform the doctor, this patient has financial difficulties, please help to consider the cost of treatment (MSW10).

MSWs functioning within the external model demonstrated proficiency in tapping into social resources beyond hospital boundaries. They achieved unique professional legitimacy through their ability to establish links with external entities such as social welfare organisations. A social worker shared the experience of gaining resources for patients in financial need. With over a decade of experience in this field, she has developed an extensive network of social resources, allowing her to access various forms of support:

Over the years, we have established a stable base of donors. Many people who wish to contribute will contact us, and some social welfare organisations deliver supplies to us by the truckload. We then integrate these items and distribute them to needy patients (MSW24).

Furthermore, the external MSWs were affiliated with third‐party organisations; they had access to extensive social resources beyond the hospital. For instance, they could organise support groups for patients outside the hospital. The next extract demonstrates how MSWs leveraged the authority of the government to elevate their professional status.

We can receive support from both government and our social organisation, which enables us to leverage governmental resources, secure additional funding to establish a ‘Unified Treatment Platform’ and invite doctors and patients from various hospitals to participate. (MSW 13)

Regarding resource mobilisation, both internal and external MSWs have highlighted a collaborative approach, meaning that the two models of social work combine their strengths to work jointly. A social worker discussed this shared platform, detailing the process of its creation and emphasised the specialised role, which is distinct from that of doctors and nurses in terms of care and treatment, serving instead as a complementary function.

We have several MSWs from different hospitals, and at the beginning, we frequently communicated with each other. Then, we thought it would be better to establish a resource‐sharing platform, which includes policies we collected, medical assistance guidelines, and policies about insurance, as well as some social welfare organizations that we can connect with (MSW28).

In their role as resource mobilisers, MSWs viewed their work as a supportive extension of healthcare but subordinate to doctors' efforts. They aim to facilitate patients' continued treatment when lacking resources. As a social worker said:

Our role is to assist doctors in providing better treatment to patients by doing what they cannot (MSW17).

Nevertheless, MSWs’ ability to mobilise resources is highly influenced by the availability of those resources; therefore, it shifts attention from individual competence to institutional and economic contexts

Greatly affected by reality, especially in the past two years, hospitals don't have money, and charitable organizations don't have money either. Sometimes, we’re powerless too (MSW28).

The two models of MSW practice demonstrate distinct strengths in resource mobilisation. Internally embedded MSWs use their understanding of hospital systems to access institutional resources, whereas externally affiliated MSWs are better at engaging wider community and social welfare networks. Our findings also reveal that the availability of social resources significantly influences MSWs' capacity to mobilise support. However, the profession currently lacks the institutional authority or structural capacity to create new charitable or welfare resources, limiting its role mainly to coordinating existing ones.

## 4 Discussion and Conclusion

This paper set out to address two questions. First, what strategies have MSWs in China adopted to gain professional legitimacy and how are these enacted in the two employment models? It further explores the broader implications of this case for understanding the emergence and legitimation of new professional groups within recently marketised welfare systems.

It should be noted that these different employment models represent distinct structural environments, which are not simply administrative arrangements but represent two distinct strategies of professional legitimation within a marketised healthcare system. This paper further defines legitimacy as the formal, top‐down recognition of the MSW's jurisdiction within the hospital, whereas trust refers to the interpersonal expectations between clinical actors, as well as between patients and healthcare institutions more broadly. Drawing on empirical findings, this study identifies three interrelated strategies through which MSWs negotiate their professional status in both models: external manifestation, alignment with the central state mandates and resource mobilisation. Table 2 summarises how three strategies operate across the two contexts.

**TABLE 2:** Legitimation strategies in the two employment models.

Strategies	Internal embedding	External collaboration
External manifestation	MSWs wear white coats. Aligning with medical staff to gain early recognition may obscure role distinction.	MSWs wear blue vests. Face initial credibility challenges but later benefit from clearer role differentiation.
Political alignment	Embedded in hospital system. Directly serve state goals (e.g., stability and dispute resolution).	Perceived as more neutral by patients. Better positioned to represent patient interests in disputes.
Resource mobilisation	Access to internal hospital funds, referrals and infrastructure. Embedded in institutional workflows.	Mobilises community and social service networks. More flexible but dependent on partnerships.

Professions use specific symbols and attire as strategies in professional identity formation (Abbott 1988). Our study observed that the two models of MSW adopted different visible markers, the white coat and the blue vest, to establish their professional legitimacy. In a healthcare environment where biomedicine dominates and institutional hierarchy is deeply entrenched (Shi 2022), the adoption of the white coat by internally embedded MSWs serves as a symbolic alignment with medical authority. This strategy proved effective in facilitating early‐stage recognition within hospital settings.

However, the findings suggest that, although the white coat enhances visibility and perceived legitimacy, it may obscure the MSW's distinct professional identity by conflating them with clinical staff. In contrast, externally affiliated MSWs, who often wear the blue vest, initially face greater challenges in establishing their presence and authority. Yet, over time, this distinct attire enables clearer differentiation from medical personnel, fostering public awareness of the MSW's unique role. These findings echo earlier observations that clothing is not merely symbolic but instrumental in professional boundary work (Timmons and East 2011).

Beyond symbolic representation, professional legitimacy in the Chinese context also hinges on responsiveness to political imperatives. Professions often gain legitimacy by aligning with the normative and instrumental goals of powerful stakeholders (Tidmarsh 2022b). MSW was re‐established in the early 2000s through state‐led initiatives to address growing medical disputes, public distrust and inequalities in healthcare access (Liu 2008). In this policy context, MSWs function not only as service providers but also as state‐aligned actors tasked with restoring trust and maintaining social stability. Demonstrating responsiveness to these political agendas is thus a foundational condition for legitimacy (Tian and Zhang 2025). The two models of MSW in this study diverge in how they meet these expectations. Internally embedded MSWs, positioned within hospital hierarchies, often serve as intermediaries between staff and patients, helping to defuse conflict from within institutional structures. In this sense, MSWs exemplify what Noordegraaf (2020) terms ‘organised professionalism’: a form of legitimacy grounded not in self‐regulation but in institutional adaptability and multi‐stakeholder alignment.

However, this alignment may compromise their perceived impartiality among patients, who may view them as serving hospital interests. Conversely, externally contracted MSWs with fewer institutional restraints often enjoy greater trust from patients and are perceived as more independent mediators. Yet this very independence can create barriers to institutional access and integration, raising concerns about sustainability and professional marginalisation.

A further strategy shaping MSW legitimacy is their capacity for resource mobilisation, which reflects their ability to fulfil unmet needs within an under‐resourced healthcare system. Professions differentiate themselves by asserting their unique expertise and highlighting their ability to perform tasks that constrain other professions (Abbott 1988; Norris 2001). Our study reflects earlier investigations of MSW regarding its supportive role and reveals that MSWs played a vital role in mobilising both institutional and external resources, including financial aid, social welfare referrals and community‐based services. These abilities differentiate MSWs from physicians, who focus primarily on ‘cure’, and nurses, who are traditionally aligned with ‘care’, establishing them as a distinct professional legitimacy that complements, rather than merely duplicates, existing medical and nursing roles. Internally embedded MSWs often had privileged access to hospital infrastructure, enabling them to coordinate internal support more efficiently. In contrast, external MSWs were typically more effective at accessing out‐of‐system resources, including welfare support and grassroots assistance. These divergent capacities illustrate how both models contribute to the profession's functional differentiation from clinical staff. Resource mobilisation not only addresses the material consequences of healthcare marketisation but also affirms the MSW's value within multidisciplinary care. However, a notable limitation remains: the current strength of MSWs lies predominantly in integrating and navigating existing resources rather than in generating new systems of support. Although this intermediary role is valuable, it underscores the need for institutional investment and supportive policy environments to enhance the profession's structural autonomy and long‐term development.

The institutional settings for the legitimation of MSWs have a major influence on the efficacy of the different strategies being used by the profession. Internal embedding is more conducive to converting legitimation claims into routine in‐hospital access and task jurisdiction, whereas external collaboration is more conducive to converting legitimation claims into cross‐boundary resource mobilisation and perceived independence.

This paper also aims to examine the broader implications of the development of MSW in China for understanding the formation of new professional groups in marketised welfare systems. The analysis demonstrates that the development of MSW is both a product of and a response to China's marketised healthcare system. Positioned at the intersection of care and governance, MSWs compensate for structural deficits generated by neoliberal reforms, particularly the rise in doctor–patient disputes and the neglect of patients' social needs. These strategies reveal how new approaches to the creation of professional legitimacy can be built through visible manifestation, political alignment and resource mobilisation. We argue that the Chinese case of medical social work provides further empirical grounding for the concept of post‐professionalism. The legitimacy of MSWs does not derive from the exclusive control of specialised knowledge but from new professional identities defined through intermediation and shared competencies endorsed by policy frameworks. MSWs use their state‐granted and efficacy‐based legitimacy to act as a ‘bridge’. Their professional work is essentially the conversion of institutional legitimacy into social trust within the clinical encounter. It moves beyond traditional models of jurisdictional closure, offering a practice‐based extension of post‐professionalism in contexts where professional formation is shaped by both market dynamics and political governance. In this sense, professional authority becomes increasingly relational and context‐dependent rather than being grounded solely in exclusive knowledge claims or autonomous professional jurisdictions. This reframing contributes to a more relational and context‐sensitive sociology of healthcare professions. This study acknowledges the limitation of not including nurses in the interview sample. Given the partial overlap in supportive care, communicative and patient‐facing practices performed by both professions, future research should explore the inter‐professional dynamics between MSWs and nurses to provide a more holistic view of professional boundary work in hospital settings. While focused on China, the findings have broader relevance for other healthcare systems undergoing marketisation, managerial reform or the erosion of professional monopolies, offering insights into the construction of legitimacy under changing institutional conditions.

## Author Contributions

**Haoyang Liu:** conceptualization, investigation, writing – original draft, methodology, validation, writing – review and editing, formal analysis, project administration, data curation, resources. **Paul A. Martin:** methodology, conceptualization, supervision, writing – review and editing.

## Conflicts of Interest

The authors declare no conflicts of interest.

## Data Availability

The data that support the findings of this study are available on request from the corresponding author. The data are not publicly available because of privacy or ethical restrictions.
